# Selective Ligands for Non-Canonical DNA Structures: Do They Have a Future in Medicinal Chemistry?

**DOI:** 10.3390/ijms231911984

**Published:** 2022-10-09

**Authors:** Manlio Palumbo, Claudia Sissi

**Affiliations:** Department of Pharmaceutical and Pharmacological Sciences, University of Padova, Via Marzolo 5, 35131 Padova, Italy

Winning the war against cancer represents a major goal currently. During the last few years, we have witnessed a fundamental increase in basic knowledge in the biochemical and molecular biological fields connected to oncology, progressively unveiling more precise and selective cancer-related mechanisms and targets to be exploited. Meanwhile, the research and development spending for pharmaceuticals increased by almost an order of magnitude [1]. Even admitting the substantial steps forward thus far made, the rate of cancer death has not decreased accordingly and remains unacceptably high [2]. Hence, the need for novel anticancer strategies directed towards yet unexplored/poorly explored targets.

The Special Issue, entitled “Selective Ligands for Non-canonical DNA Structures: Do They Have a Future in Medicinal Chemistry?”, aimed to examine the therapeutic opportunities and drawbacks exhibited by ligands designed to selectively bind non-canonical nucleic acid structures, such as G-quadruplexes (G4) and highly polymorphic guanine-rich structures, participating in telomere protection, gene expression regulation, and genomic instability induction [3,4].

## 1. G4 Targeting Opportunities and Challenges

The paper by Folini and colleagues [5] offers a thoroughly updated critical view on the possibility of targeting of G4 in cancer, considering the vast repertoire of G4 binders (over 1000) as compared to the paucity of clinical trials (only two) started 6 years ago, the results of which are not available at present. The paper considers the issues of multiple mechanisms of action, synthetic lethality targeting, and adaptive responses. The conclusion follows that several challenges need to be addressed to obtain pharmacologically relevant G4 binders. These include high selectivity in target recognition, accurate unified screening procedures, and rational modifications of the molecular scaffold to grant both efficient binding and favorable pharmacokinetic and toxicity (ADMET) properties.

G4s represent conserved features in the evolution tree, which strengthens the idea of a vital role played in biological systems, including viruses. Hence, not only can anticancer agents be targeted at G4, but also antiviral compounds. Richter and colleagues [6] carefully investigated several DNA and RNA viruses, showing G4 regions both in the genome and mRNA. The addition of G4 ligands produces modulatory effects depending upon the type of virus and ligand structure. It should be mentioned that the tested ligands did not show elevated levels of G4 preference, being able to bind off-target double helical sequences as well, which are largely prevalent in the genome. The observed biological effects included promoter activity modulation, virion trapping, decreased or inhibited genome replication, and specific gene expression modulation. The authors conclude that G4 targeting could represent a valid approach to manage viral infections. The challenge here is to improve ligand selectivity for viral G4s to avoid serious side-effects arising from interference with human G4s.

## 2. Base-Modified Nucleotides

In their contribution, Lee and colleagues [7] describe the structural effects observed by chemical modification of nucleotides. Methyl, Br, and aryl substituents were incorporated into purine C8 and pyrimidine C5 positions. When introduced into a DNA chain, these modifications affect the local conformational properties and may help us understand the effects of covalent base alterations in genetic and epigenetic processes.

## 3. Selective G-Quadruplex Ligands

As mentioned before, this topic is quite popular, but, until today, no really selective agents were described. In principle, it should be possible to efficiently discriminate among G4s as they are generally inserted in different sequence context, adopt precise G4 conformational state(s), and exhibit sequentially distinct intervening loops. However, different conformations are separated by small energy barriers and can easily interconvert. Finally, the flat positively charged scaffold of the ligands can partially intercalate, with the double helical portion of DNA acting as a “sink” preventing specific G4-related effects by mass action.

Among new ligands, a group of bis-triazolyl pyridines was synthesized and investigated by Di Porzio and colleagues [8]. The design of these molecules fulfilled three requirements: a planar aromatic system for stacking purposes, a V-shaped form to maximize interactions with G4, and two or three positively charged groups to reinforce binding. Biophysical studies on G4s are generally performed using powerful spectroscopic techniques, such as circular dichroism, NMR, and fluorescence measurements to facilitate the identification and quantitative determination of G4 species present in complex mixtures. A multivariate analysis considering G4/iM (intercalated Motifs C-rich quadruplex DNA) modulation indicated two derivatives, able to stabilize G4 while destabilizing iM, which deserve further investigation.

## 4. RNA-Based G4s

Marzano and colleagues [9] show that the pharmacologically significant targeting of G4 ligands can also be applied to RNA-containing species, such as TERRA, a tetraplex-folded sequence modulating telomerase activity, heterochromatin formation, and homologous recombination. Virtual screening methods in tandem with experimental testing allowed compound BPBA to be identified, consisting of two benzimidazole moieties connected through an aniline residue as a very efficient G4-RNA binder. Biological studies in cell systems confirm the chemical results in vitro by showing specific interference with TERRA activity. It should be noted that TERRA interacts with telomeric chromatin, forming a hybrid DNA-RNA quadruplex. Analytical studies are performed by means of the above-mentioned spectroscopic techniques. 

## 5. Fluorescence Measurements

Nowak-Karnowska and colleagues [10] measured the fluorescence of 9-methoxy luminarine in the presence of G4-forming sequences. Substantial signal quenching was found in the presence of the parallel c-MYC G-quadruplex, possibly because of stacking interactions which involve the planar aromatic region of interacting species. The test ligand cannot induce G4 formation or stabilize preformed G4 structures. The above properties suggest the use of fluorescent measurements using 9-methoxy luminarine to preliminarily assess parallel vs. non-parallel G4 topologies.

## 6. NMR Studies

Using NMR and modeling techniques, Dallavalle and colleagues [11] investigated the binding of Curaxins, in particular CBl0137, to non-canonical DNA structures. This was originally not included in the proposed mechanisms of action, but recent findings seem to support G4 binding as well. To confirm this hypothesis, NMR studies were performed with the human telomere and the c-MYC promoter sequences. In both cases, curaxin was bound to G4s, forming two types of complexes with 1 or 2 ligands bound per oligonucleotide unit. Moreover, curaxin intercalates into double-stranded DNA, demonstrating poor binding selectivity. The real role of drug binding to G4 structures in the presence of double-stranded DNA requires competition measurements to assess drug distribution between G4 and B form.

Continuing with NMR spectroscopy, Krafčík and colleagues [12] discuss the in-cell technique to examine the quantitative binding of low-molecular-weight ligands with nucleic acids, enabling a high-resolution readout on structure and interactions of targeted species. This technique is highly valuable as the measurements are made at physiologically relevant conditions. Unfortunately, the in-cell ^1^H study is hardly applicable to polymorphic G4s and their complexes due to the substantial broadening and overlapping of resonance peaks. To overcome this drawback, G4 constructs were labelled with a 3,5-bis(trifluoromethyl)phenyl tag. The use of ^19^F-detected in-cell NMR may thus represent a valuable methodology to be applied in profiling G4–ligand interactions in vivo.

## 7. Conclusions

Despite the huge amount of work carried out on G4 binders, there is still no resolute answer to the original question on whether agents targeted at non-canonical nucleic acid sequences will eventually become effective and safe drugs. In fact, several issues (see also above) should be more comprehensively approached and thoroughly dissected.

- Design ligands with higher selectivity for a given G4 arrangement taking advantage of the local environment and the nature (sequence, orientation, and length) of the connecting loops. The charged nature of these molecules might not afford the best conditions for selectivity, considering that cationic binders will exhibit non-negligible electrostatic binding affinity for the canonical double helical form too.
- Find a rationale for discriminating among the various non-canonical nucleic acid conformations by test ligands.
- Quantitate canonical vs. non-canonical binding distribution within a cell, considering the large prevalence of B-DNA at pharmacologically relevant conditions
- Make sure that the experimental setting in vitro does not create artifacts by stabilizing species not occurring in vivo.
- Define standard protocols of investigation to properly compare results from different labs, with a particular focus on identifying a few reference nucleic acid sequences to be used.
- Develop artificial intelligence algorithms to unveil particular ligand features conferring high specificity and selectivity.
- Make sure to fulfil ADMET requirements prior to implement costly and time-consuming synthetic efforts.
- Consider the possibility of simultaneous recognition by the binder of two or more G4 arrangements close in space, conferring a higher degree of selectivity.
- Do not neglect the kinetic aspects of the binding, which might discriminate fast-forming species from slowly assembling structures.

Currently, the road to success appears challenging since much work (and time) is required to contend with the basic problems. However, it is worth mentioning that immunotherapy drug development took a few decades of poorly successful efforts before producing blockbuster checkpoint inhibitors. Hence, we are confident in predicting a way to rationally transform a specific G4 ligand into a real drug in a not-too-distant future.
